# Dietary habits and plasma lipid concentrations in a general Japanese population

**DOI:** 10.1007/s11306-024-02087-1

**Published:** 2024-03-05

**Authors:** Mitsuharu Sato, Eiji Hishinuma, Naomi Matsukawa, Yoshiko Shima, Daisuke Saigusa, Ikuko N. Motoike, Mana Kogure, Naoki Nakaya, Atsushi Hozawa, Shinichi Kuriyama, Masayuki Yamamoto, Seizo Koshiba, Kengo Kinoshita

**Affiliations:** 1 Tohoku Medical Megabank Organization, Tohoku University, 2-1, Seiryo-machi, Aoba-ku, Sendai, Miyagi 980-8573 Japan; 2 Advanced Research Center for Innovations in Next-Generation Medicine, Tohoku University, 2-1, Seiryo-machi, Aoba-ku, Sendai, Miyagi 980-8573 Japan; 3 Laboratory of Biomedical and Analytical Sciences, Faculty of Pharma-Science, Teikyo University, 2-11-1, Kaga, Itabashi-ku, Tokyo, 173-8605 Japan; 4 Graduate School of Medicine, Tohoku University, 2-1 Seiryo-machi, Aoba-ku, Sendai, Miyagi 980-8575 Japan; 5 International Research Institute of Disaster Science, Tohoku University, 2-1 Seiryo-machi, Aoba-ku, Sendai, Miyagi 980-8573 Japan; 6 Graduate School of Information Sciences, Tohoku University, 6-3-09 Aramaki Aza-Aoba, Aoba-ku, Sendai, Miyagi 980-8579 Japan

**Keywords:** Metabolomics, Dietary lipids, Plasma lipids, Dairy intake, Seafood intake, Odd-chain fatty acids, Sphingomyelin, Omega-3 fatty acids, Hydroxy fatty acid

## Abstract

**Introduction:**

Accumulating data on the associations between food consumption and lipid composition in the body is essential for understanding the effects of dietary habits on health.

**Objectives:**

As part of omics research in the Tohoku Medical Megabank Community-Based Cohort Study, this study sought to reveal the dietary impact on plasma lipid concentration in a Japanese population.

**Methods:**

We conducted a correlation analysis of food consumption and plasma lipid concentrations measured using mass spectrometry, for 4032 participants in Miyagi Prefecture, Japan.

**Results:**

Our analysis revealed 83 marked correlations between six food categories and the concentrations of plasma lipids in nine subclasses. Previously reported associations, including those between seafood consumption and omega-3 fatty acids, were validated, while those between dairy product consumption and odd-carbon-number fatty acids (odd-FAs) were validated for the first time in an Asian population. Further analysis suggested that dairy product consumption is associated with odd-FAs via sphingomyelin (SM), which suggests that SM is a carrier of odd-FAs. These results are important for understanding odd-FA metabolism with regards to dairy product consumption.

**Conclusion:**

This study provides insight into the dietary impact on plasma lipid concentration in a Japanese population.

**Supplementary Information:**

The online version contains supplementary material available at 10.1007/s11306-024-02087-1.

## Introduction

The metabolome serves as an intermediary phenotype linking lifestyle and phenotype, responding to factors such as diet, disease, aging, and lifestyle changes. Lipids comprise a part of the metabolome and play essential roles in human health; however, these species can also contribute to lifestyle diseases. Associations between food consumption habits and blood lipid levels have been investigated in various populations. In the Exposome-Explore database (http://exposome-explorer.iarc.fr), as of April 21, 2022, a total of 3431 significant correlations between food or nutrient consumption habits and lipids are available based on 123 studies. These studies include countries such as the USA (41 reports); the UK (11 reports); Sweden, Norway, and Japan (seven reports); and Spain and Australia (six reports). Lipids targeted in these studies mainly comprise fatty acids (FA) (1639/3431) and organic pigments (1791/3431). Of the 1639 FA associations reported to date, the largest number relate to omega-3 FA (516 associations, 31.4%), saturated/monounsaturated FA (415 associations, 25.3%), odd-carbon-number FA (odd-FA; 235 associations, 14.3%), and omega-6 FA (164, 10.0%). The database does not include postprandial changes in blood lipid levels or results from animal experiments.

Several lipid biomarkers of dietary habits have been extensively studied. For example, the association between seafood consumption and omega-3 FA has been investigated in a number of populations, including those from Japan (Okuda et al., 2009; Wakai et al., 2005), China (Zhang et al., 2010), Europe (Turunen et al., 2010), the USA (Sublette et al., 2011), and Australia (Mina et al., 2007). While associations between dairy consumption and odd-FA have been reported in Europe (Sweden (Wennberg et al., 2009; Wolk et al., 2001) and Norway (Sofie Biong et al., 2006)) and Oceania (New Zealand (Benatar & Stewart, 2014; Golley & Hendrie, 2014; Meikle et al., 2015) and Australia (Golley & Hendrie, 2014; Meikle et al., 2015)), there are no reports in an Asian population, to date. Although odd-FAs comprise well-known biomarkers of dairy consumption, whether these lipids are associated with dairy consumption in Asian populations remains unclear.

Gaining new insights into the processes of digestion and absorption is essential for understanding the association between diet and the plasma levels of FA-containing lipids, including the lipid subclasses targeted in this study. Although different mechanisms exist for each lipid subclass and FA type, most lipid subclasses targeted in this study are digested into constituent components which are reassembled after absorption. Thus, when the levels of a lipid species are found to correlate with one kind of food consumption, all components containing that lipid are candidate nutrients for the cause of the association. Importantly, reassembly after absorption does not necessarily reconstruct the same lipid that was digested. For example, lysophosphatidylcholine acyltransferase 3, a major enzyme in the small intestine that forms phospholipids (PL), including phosphatidylcholine (PC) and phosphatidylethanolamine, from lysophospholipids, has a substrate preference for lysophosphatidylcholine (LPC) with a saturated fatty acid at the sn-1 position and favors a polyunsaturated FA acyl donor for the sn-2 position (Kazachkov et al., 2008).

To clarify the associations between lifestyle factors and the metabolome, various influencing factors should be measured in parallel. In the present study, omics research conducted as part of the Tohoku Medical Megabank Community-Based Cohort Study (TMM CommCohort Study) aimed to satisfy this requirement (Koshiba et al., 2018; Kuriyama et al., 2016). To this end, plasma samples were collected in parallel with a food frequency questionnaire (FFQ), and omics analyses of the samples were performed in a managed environment to reduce environmental noise. We aimed to conduct a cross-sectional correlation analysis of daily consumption in 15 food categories and 439 plasma lipid species using data from 4032 Japanese individuals and validate significant correlations covering known biomarkers and new associations to provide insights into diet–lipid associations across various lipid subclasses.

## Materials and methods

### Ethical approval

This study was part of the Tohoku Medical Megabank Organization Omics study, which was approved by the Ethics Committee of Tohoku University. All the participants provided written informed consent.

### Participant selection

**Fig. 1:**
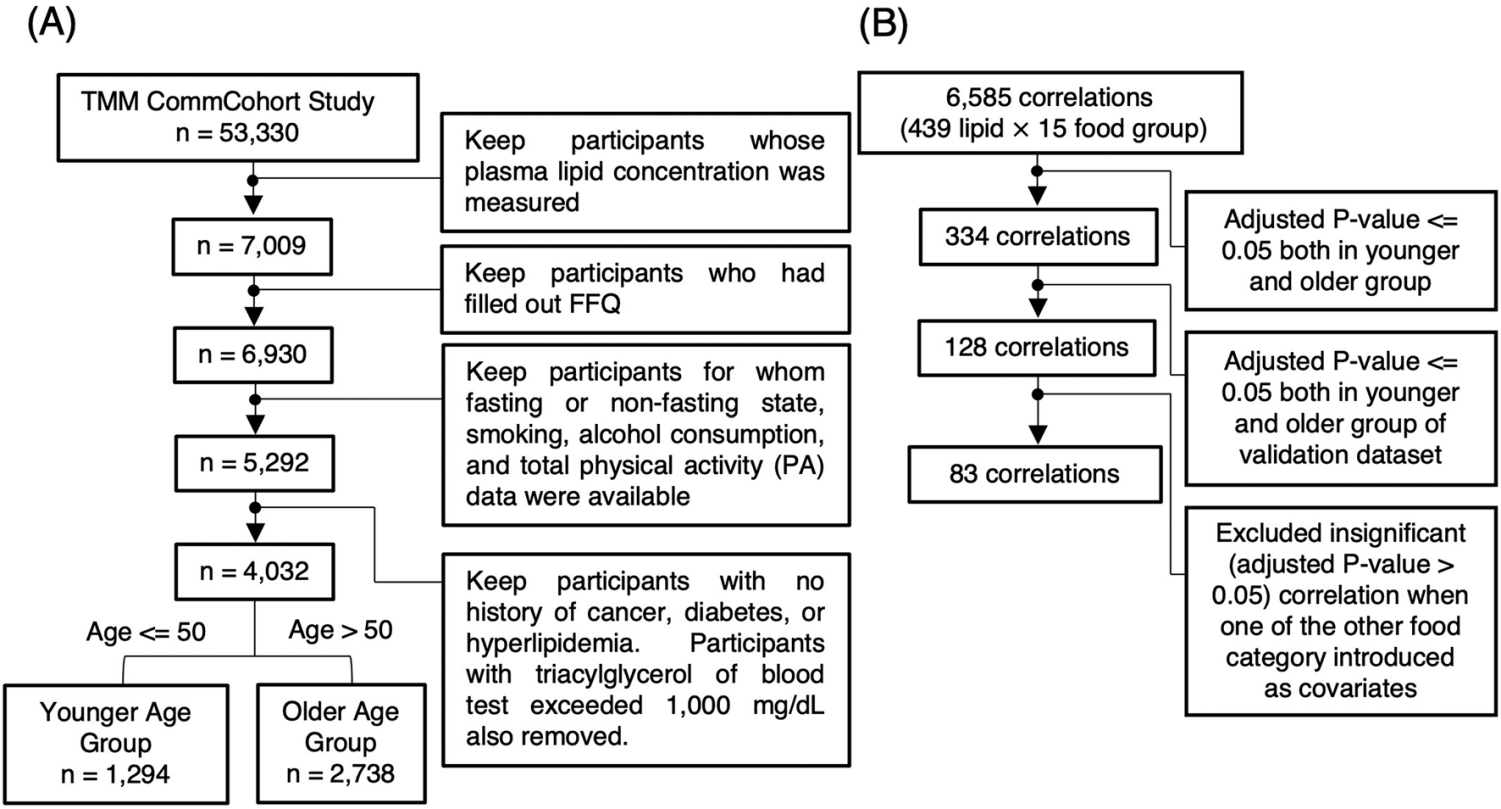
Schemes of participant selection and correlation calculation. **A** Participant selection was initiated from the entire cohort of the TMM CommCohort Study, which included 4032 participants. **B** Scheme of significant correlation selection. A total of 6,585 food-lipid associations was reduced to 128 associations by thresholding the adjusted *p*-value in each age group as well as in the validation dataset. Among them, 84 associations were still significant when any food category was introduced as an additional covariate. These 83 associations were considered as significant and independent associations in this study

This cross-sectional study aimed to evaluate an omics panel in the TMM CommCohort Study and explore new lipid biomarkers for food consumption in a general Japanese population. For these purposes, we selected samples from non-pregnant participants in the survey of the TMM CommCohort Study with the following inclusion criteria (Fig. 1A): (1) participants with plasma lipid concentration measured using mass spectrometry (N = 7009); (2) participants who had filled out the FFQ (N = 6930); (3) participants for whom fasting or non-fasting state, smoking, alcohol consumption, and total physical activity (PA) data were available (N = 5292); and (4) participants whose blood triacylglycerol levels were less than 1000 mg/dL, with no history of cancer, diabetes, or hyperlipidemia (N = 4032). Plasma lipid concentrations were measured for an additional 1493 participants from the same cohort and were used for the validation study.

### Measurements

The daily consumption in each food category was predicted using a self-reported FFQ consisting of 138 food and beverage items. The FFQ was originally developed for the Japan Public Health Center-based Prospective Study (Tsubono et al., 1996; Watanabe et al., 2001) and modified by Takachi et al. (2011) to cover urbanized Japanese populations. A unique response option for intake frequency “constitutionally unable to consume” for individual food items was added to cover the case wherein a participant is unable to eat food items due to allergies or other symptoms. The daily consumption for each food item was calculated by multiplying the median intake frequency by the intake portion size. Participants selected intake frequency from 10 options for each food item: “constitutionally unable to consume,” “never or less than once a month,” “1 to 3 times/month,” “1 to 2 times/week,” “3 to 4 times/week,” “5 to 6 times/week,” “once a day,” “2 to 3 times/day,” “4 to 6 times/day,” and “more than 7 times/day,” and also chose the usual portion size from three options; “less than half the standard portion size (× 0.5),” “same as the standard portion size (× 1),” or “more than 1.5 times the standard portion size (× 1.5).” A standard portion size was given for each food item in the questionnaire. Consumption in each food category was calculated by summing the intake volume of food items belonging to that food category. Daily consumption volumes in 15 food categories, comprising meat, dairy, eggs, seafood, vegetables, pickles, fruits, cereals, confectionery, pulses, nuts and seeds, potatoes, mushrooms, seaweed, and fats and oils, were calculated. The attribution of each food item to a food category and the calculation of daily energy intake were performed following Takachi et al. (2011) and the Standardized Tables of Food Composition, Fifth Revised Edition (Resource Council et al., 2002).

Smoking status (smoker or not), alcohol consumption (drinker or not), and total PA (kcal/day) were recorded from the responses to a self-reported questionnaire. Non-smokers were defined as never smokers, ex-smokers, or smokers whose total cigarette consumption across their lifespan was less than 100. Non-drinkers were participants who had never drunk alcohol or were ex-drinkers. Participants with conflicting answers were excluded. Total PA was calculated following Kikuchi et al. (2020), based on questions regarding occupational activities (including household and transportation), leisure time activities, and sleeping hours. Total PA was calculated as the sum of PA for occupational activity, leisure time activity, sleeping, and other activities, as shown below:

$${\text{PA }}\left( {{\text{total}}} \right) = {\text{PA }}\left( {\text{occupational activity}} \right) + {\text{PA }}\left( {\text{leisure time activity}} \right) + {\text{PA }}\left( {{\text{sleeping}}} \right) + {\text{PA }}\left( {{\text{other}}} \right)$$

For occupational activity, the participants selected the number of hours spent at four different activity levels (sitting, standing, walking, and strenuous work) on a typical day during the past year. For leisure activities, participants selected the frequency and number of hours of each of the four different leisure activity levels (walking slowly, walking quickly, light to moderate exercise, and strenuous exercise) during the past year. Sleeping hours were also recorded, and the time for “other activities” was calculated by subtracting the sum of occupational, leisure, and sleep time from 24 h. Participants who had negative values for other activity times were assessed as “zero” for other activities. The assigned metabolic equivalents of tasks (METS) for the four different occupational activity levels, which consisted of sitting, standing, walking, and strenuous work, were 1.3, 2.0, 3.0, and 6.0, respectively. For leisure time activities, the METS assigned for walking slowly, walking quickly, light to moderate exercise, and strenuous exercise were 2.8, 4.0, 3.0, and 6.0, respectively. Values of 0.9 METS and 1.3 METS were assigned to sleep and other activities, respectively. The PA for each activity was calculated by multiplying the assigned METS by the number of hours spent on each activity.

Plasma lipid concentrations were measured as described previously (Hishinuma et al., 2021; Saigusa et al., 2021). Briefly, the plasma lipid concentrations were measured using targeted metabolomics following the manufacturer’s instructions for the MxP Quant 500 Kit (Biocrates Life Sciences AG, Innsbruck, Austria) with different UHPLC-MS/MS systems. Data of 2480 participants were obtained using a Xevo TQ-S system (Waters, Milford, MA), while data of 3067 participants were obtained using a Xevo TQ-XS MS/MS system (Waters) in flow injection analysis mode. In total, 439 lipids across nine subclasses were measured. The following lipid classes were measured: TG, diacylglycerol, PC, LPC, PlsCho, SM, Cer, cholesterol esterCE, and FA. Potential isobars and isomers within 0.5 Da of the annotated formula were assessed using LIPID MAPS (https://www.lipidmaps.org).

### Quantification and statistical analysis

As the participants in this study had a broad range of ages, and as menopause is known to substantially alter lipid metabolism, participants were categorized into two groups by age, based on the average menopausal age (50 years) in the Japanese population. Among the females whose menopausal information was available, 6.5% of females aged 50 years old or younger were postmenopausal, and 2.1% of females older than 50 were premenopausal. The menopausal information of 91 females was not available. Considering this data, 50 years old was considered an acceptable threshold for dividing the cohort. A partial Spearman’s rank-sum correlation was employed to calculate and record the correlation coefficient; age, body mass index (BMI), sex, non-fasting/fasting, smoking status, drinking status, total PA, and interval for storage in the biobank were considered as covariates. Most samples were stored in the biobank on the day of sampling; however, 51 samples had an interval of one day from blood collection to storage in the biobank. The interval for storage in the biobank was represented by a dummy variable of 0 or 1, named DateDiff. Sex, non-fasting/fasting status, smoking status, and drinking status were introduced using binary values. The adjusted *p*-value estimated following the Benjamini and Hochberg (Benjamini & Hochberg, 1995) method was used to adjust for multiple tests. For correlations of food category consumption, partial Spearman’s rank correlation coefficients were calculated, with age, BMI, sex, drinking status, smoking status, PA, and daily energy intake as covariates.

To obtain an overview of the correlations, we used ComplexHeatmap 2.10.0 (Gu et al., 2016). For the calculated correlation coefficients among lipids, a partial Spearman’s rank-sum correlation was used with covariates (age, sex, BMI, non-fasting/fasting, drinking/non-drinking, smoking/non-smoking, total PA, and DateDiff). The relationships between lipids were inferred by hierarchical clustering using Ward’s D2 method with the hclust function in R 4.1.2 (R Core Team, 2021). The distance matrix is the correlation coefficient between the lipids subtracted from one.

## Results

### Characteristics of participants

The characteristics of the participants in the discovery dataset are listed in Table 1. This study divided participants into two datasets according to age (above and below 50 years). A total of 1297 participants (287 males and 1010 females) were included in the younger age group, while 2751 participants (1080 males and 1671 females) were included in the older age group. In both age groups, we verified that the blood concentrations of triacylglycerol and glucose were lower in the fasting group when compared to the non-fasting group.

**Table 1:** Characteristics of study participants

	Younger age group				Older age group			
	Male		Female		Male		Female	
Postprandial state	Fasting	Nonfasting	Fasting	Nonfasting	Fasting	Nonfasting	Fasting	Nonfasting
N	95	192	421	589	317	763	610	1060
Age	39.4 (6.5)	40.7 (6.6)	40.8 (6.9)	39.9 (6.6)	66.6 (6.2)	66.9 (6.1)	63.3 (6.9)	63.8 (6.9)
BMI	23.2 (3.5)	23.4 (3.2)	21.7 (3.6)	21.8 (3.7)	24.1 (8.7)	23.7 (2.6)	22.3 (3.2)	22.4 (3.3)
DateDiff = 1	0	0	0	0	2	25	2	22
Physical activity per day (kcal)	2786 (1213)	2505 (862)	2290 (573)	2367 (749)	2456 (685)	2584 (877)	2432 (668)	2455 (735)
Smoking (%)	31.6	32.3	10.7	13.2	14.8	19.1	3.9	4.4
Triacylglycerol (mg/dL)	116.7 (107.4)	149.2 (117.4)	71 (39)	82.4 (49.9)	114.1 (67.5)	126.2 (83.1)	91 (43.8)	110.5 (62.4)
Glucose (mg/dL)	84.2 (11)	88.2 (15.8)	80.5 (8)	84 (12.6)	91 (12.9)	95.8 (18.9)	84.7 (8.1)	89.4 (15.1)
Drinking (%)	71.6	72.4	50.6	49.1	77	80.2	46.9	43.7
Energy intake per day (kcal)	2285.7 (229.8)	2264.8 (249.3)	1834.7 (97.6)	1843.7 (110.8)	2285.2 (236.1)	2284 (228.9)	1866.6 (132.3)	1859.5 (113.9)
Meat	101.7 (96.6)	89.4 (78.7)	75.9 (60.9)	84.1 (141.5)	73.7 (100.3)	68.9 (57.8)	80.4 (110.8)	68.5 (81.3)
Dairy	212.5 (368)	162.6 (233)	238.4 (421.1)	231.4 (352.7)	260.7 (352.2)	248.8 (395.1)	324.4 (469.4)	318.3 (425.9)
Eggs	28.7 (31.4)	28.6 (35.1)	31.7 (50.6)	30.3 (45.1)	32.6 (48.5)	31.2 (43.1)	34.1 (54)	34.1 (53.3)
Seafood	52.2 (38.9)	46.2 (40.8)	44.7 (35.3)	52.4 (61.4)	67.8 (60.8)	75.5 (105.8)	79.9 (117.3)	76.1 (86.2)
Vegetables	167.7 (160)	154.2 (137.7)	196.4 (157)	209.1 (187.4)	227.9 (175.4)	226.1 (185.9)	303 (239.1)	300.9 (241.1)
Pickles	11.6 (12.6)	13.5 (19.9)	13.7 (27.7)	15.2 (34.1)	26 (35.8)	27 (39.7)	30.5 (54.8)	29.2 (43.2)
Fruits	89.3 (95.6)	127.2 (334.2)	142.1 (213.9)	154.1 (236.1)	177.9 (196.9)	170.7 (183.8)	248.8 (272.7)	243.2 (212.9)
Cereals	606 (271.8)	565.8 (194.3)	416.2 (181.5)	436.6 (205)	558.6 (187.5)	549.1 (194.3)	419.8 (240.5)	422.1 (201.6)
Confectionery	14.7 (16.1)	21 (32.3)	26.8 (33.1)	24 (28.7)	17.8 (21.3)	17.1 (23.2)	28.8 (32.3)	28.8 (52)
Pulses (g/day)	51.7 (45.1)	63.8 (85.2)	84.2 (130.8)	88.9 (151.7)	89.5 (104.1)	92.9 (105.8)	112.3 (132.6)	113.8 (144.7)
Nuts and seeds	1.1 (1.9)	3 (11.9)	1.3 (2.6)	1.5 (3.3)	2.9 (6)	3.1 (8.6)	3.3 (10.8)	3.3 (9.7)
Potatoes	24.8 (29.8)	21 (21.6)	32.4 (72.9)	30.2 (34.6)	33.3 (28.4)	33.4 (36.2)	44.5 (40.8)	44.7 (45.2)
Mushrooms	7.3 (7.9)	7.8 (11)	12.4 (18.3)	13.2 (14.7)	10.2 (10.6)	10.9 (13.6)	18.6 (24.8)	17.9 (30.6)
Seaweed	6.5 (5.8)	7.7 (9.2)	8.5 (15.3)	8.5 (10.4)	10.1 (9.5)	10.5 (11.7)	11.6 (11.1)	11.1 (14.6)
Fats and oils	11.8 (8.4)	10.5 (6.8)	11.6 (8.4)	12.8 (12.4)	11.6 (8.7)	12.1 (8.3)	14.9 (12.5)	14.3 (10.9)

Values for standard deviation of the mean are given in parentheses

The unit for food consumption is g/day

### Dietary correlations were identified and subsequently validated by the removal of pseudo-correlations

The consumption of most food categories was positively correlated each other, other than cereal consumption (Online Resource 2). All categories, except nuts and seeds, showed a bell-shaped distribution, along with the presence of some non-consumers (Online Resource 1). To remove pseudo-correlations among food category consumption, all correlations between food consumption and plasma lipid concentration were recalculated by adding consumption in other food categories singly as covariates, retaining only correlations that remained significant when controlled for consumption of any other food category (Fig. 1B).

Given that the design of this study was not suitable for evaluating differences between age groups as both food consumption habits and lipid metabolism change across age groups, we focused on correlations that were significant in both age groups. The selection steps for significant correlations are illustrated in Fig. 1B. Correlations were validated between age groups and between the discovery and validation datasets, resulting in 128 preliminary correlations. To remove pseudo-correlation among food categories, only those correlations that were still significant when all other food categories were introduced singly as covariates were retained. By this means, 84 correlations were validated and considered independent of different food categories. Lipids correlated with the consumption of each food category are summarized in Table 2.

**Table 2:** Lipids correlated with food categories

Food category	Lipids positively correlated	Lipids negatively correlated
Seafood	PC aa C36:0, PC aa C36:5, PC aa C36:6, PC aa C38:0, PC aa C38:5, PC aa C38:6, PC aa C40:3, PC aa C40:6, PC aa C42:0, PC aa C42:1^a,c^, PC aa C42:2, PC aa C42:5, PC aa C42:6, PC ae C30:1, PC ae C32:2, PC ae C34:0^a,b,c^, PC ae C36:0, PC ae C38:0, PC ae C38:6, PC ae C40:2^c,d^, PC ae C40:5, PC ae C40:6, SM C26:1, DHA, EPA, Cer(d18:1/26:1)^a,b,c^, CE(20:5), CE(22:5)^a,b,c,d^, CE(22:6), TG(16:0_38:6), TG(16:0_40:7), TG(16:0_40:8), TG(18:0_38:6)^a,b,c,d^, TG(18:1_38:6), TG(18:1_38:7), TG(18:2_38:6), TG(20:5_34:1), TG(20:5_34:2), TG(20:5_36:2), TG(20:5_36:3), TG(22:5_34:1)^a,b,c,d^, TG(22:5_34:2)^c^, TG(22:6_32:0), TG(22:6_32:1), TG(22:6_34:1), TG(22:6_34:2), TG(22:6_34:3)	PC aa C36:3
Confectionery	PC ae C34:2^a,b,c^, PC ae C36:2^d^, PC ae C36:3^a,b,c^, SM(OH) C14:1^a,b^, Cer(d18:1/22:0)^b,c^, Cer(d18:2/22:0)^a,c^, Cer(d18:2/24:0)^a,b,c^, Hex3Cer(d18:1_22:0)^a,b,c^	lysoPC a C16:1, PC aa C38:6, PC aa C40:6, PC aa C42:5^c^, PC ae C38:0^d^, TG(16:0_38:6)^d^, TG(16:0_38:7)^a,c,d^, TG(16:1_38:5), TG(18:1_38:6), TG(18:1_38:7)^d^, TG(18:2_38:6)^d^, TG(20:5_34:1)^d^, TG(22:6_32:0)^d^, TG(22:6_34:1), TG(22:6_34:2)^d^
Dairy	lysoPC a C17:0, PC ae C30:0, PC ae C36:2^d^, SM(OH) C14:1, SM(OH) C16:1, Cer(d16:1/23:0)^d^	
Meat	PC ae C36:4, PC ae C38:4^a^, PC ae C38:5^a^	
Cereals	TG(16:1_34:3)^a,c^	PC aa C28:1^a,d^
Fats and oils		PC aa C32:1

^a^Not significant in at least one sex when participants were stratified by sex in the older age group

^b^Not significant in at least one age group when participants were stratified by age in the female group

^c^Not significant in at least one of fasting or non-fasting participants when participants were stratified by fasting/non-fasting in the older age group

^d^Not validated as significant via the correlation selection scheme shown in Fig. 1B when postmenopausal younger age females and premenopausal older age females were removed from the analysis

**Fig. 2:**
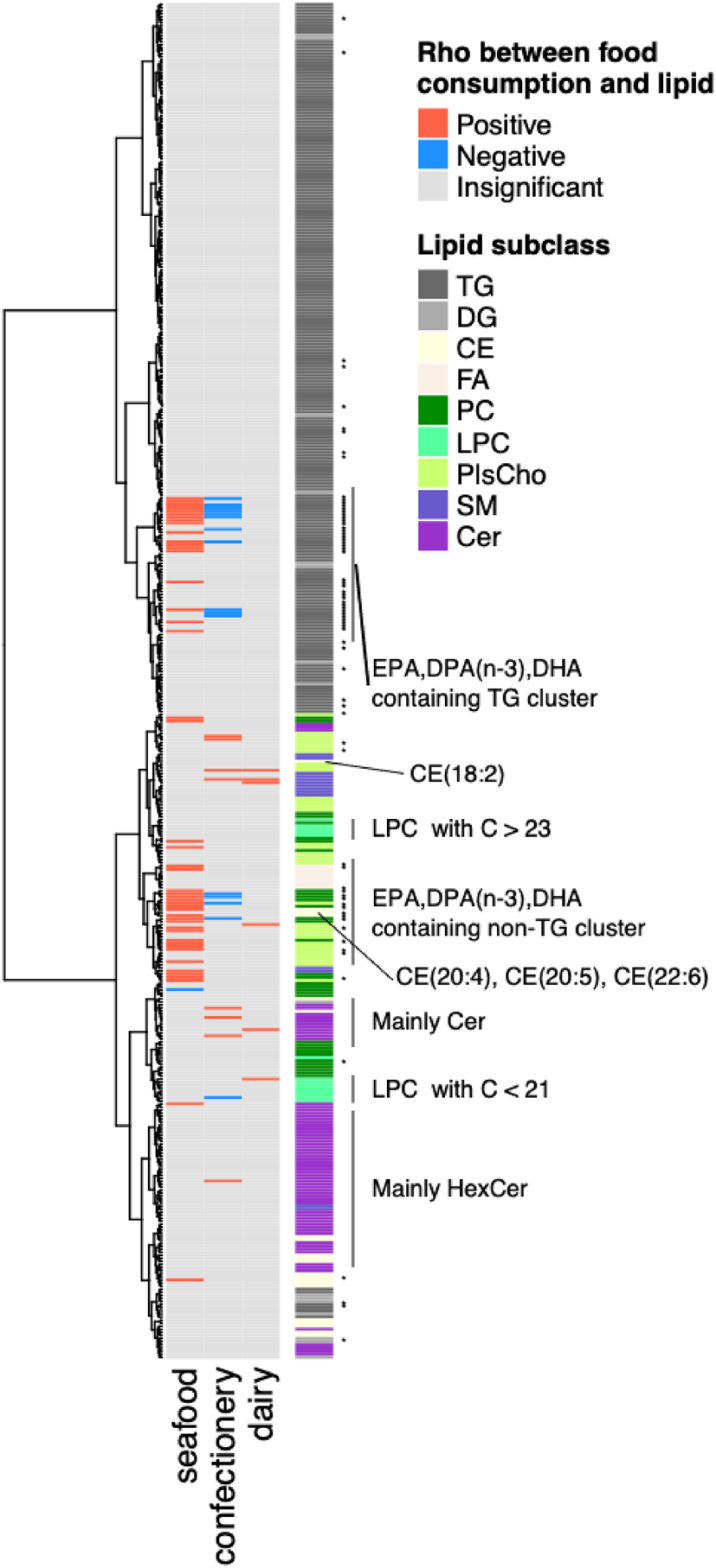
An overview of correlations between food category consumption and the concentrations of plasma lipids The columns of the heatmap are food categories. Lipid species are aligned in the row by the order of the dendrogram based on correlation coefficients among lipids. Significantly positive and negative correlations between food category consumption and lipid concentration are indicated by red and blue boxes, respectively. Insignificant correlations are shown by gray boxes. The lipid subclass for each lipid is indicated by colored boxes in the right side of the heatmap. The colors are dark gray, triacylglycerol (TG); light gray, diacylglycerol (DG); light yellow, cholesterol ester (CE); linen, fatty acid (FA); green, phosphatidylcholine (PC); sea green, lysophosphatidylcholine (LPC); olive green, choline plasmalogen (PlsCho); slate blue, sphingomyelin (SM); and purple, ceramide (Cer). Lipids possibly containing EPA, DPA(omega–3), and DHA are shown by the asterisks

Understanding relationships between lipids is essential for discussing dietary factors that influence plasma lipid concentrations. We mapped 84 food–lipid correlations using hierarchical cluster analysis based on the distance matrix generated by subtracting the correlation coefficient among lipids from one (Fig. 2). A dendrogram showed that the concentration of lipids varied with the lipid subclass. Lipids could be divided into triglyceride (TG) and non-TG clusters. In the non-TG group, FA, ceramides (Cer), sphingomyelin (SM), and hexosylceramides (HexCer) clustered further, with some exceptions. In contrast, phosphatidylcholine (PC) and choline plasmalogen (PlsCho) species did not form a cluster. The distribution of lipids in the dendrogram was also affected by FA type. LPCs fell into two clusters, depending on the carbon number of the FA. Lipids containing 20:5, 22:5, and 22:6 FA, assumed to comprise omega-3 eicosapentaenoic acid (EPA)-, docosapentaenoic acid (DPA)-, and docosahexaenoic acid (DHA)-containing lipids, and lipids containing FA with more than five double bonds clustered in TG and non-TG groups. Considering this correlation among lipids, we discuss associations with the food categories of seafood, confectionery, and dairy products.

### Seafood consumption has marked effects on plasma lipids

Seafood consumption correlated positively with 60 lipids and negatively with PC diacyl (aa) C36:3 (Table 2). Regarding omega-3 FA, a well-known biomarker of seafood consumption, EPA and DHA were positively correlated with seafood consumption. In addition to omega-3 FA, cholesterol ester (CE containing 20:5, 22:5 and 22:6) showed positive correlations, with the constituent FA species expected to include EPA, DPA, and DHA, respectively. Similarly, all positively correlated TGs contained 20:5, 22:5, 22:6, or more than six double bonds in two unknown FA species. These TGs formed a cluster in the dendrogram (Fig. 2), suggesting that the TG group containing EPA, DPA, and DHA correlated with seafood consumption. Approximately half of the positively correlated glycerophospholipids (GPLs) were highly unsaturated. However, the rest were not, including PC aa C36:0 and PC acyl-alkyl (ae) C34:0. The hierarchical cluster analysis for lipids suggested that all GPLs, except for PC aa C42:0 and PC aa C42:1, contained EPA, DPA, or DHA, or were related to omega-3 FA, as these species clustered into EPA-, DPA-, and DHA-containing non-TG clusters. Apart from omega-3 FA, SM C26:1, Cer (d18:1/26:1), PC aa C42:0, and PC aa C42:1 were positively correlated with seafood consumption (Table 2). As an additional evaluation of the correlation between metabolites and seafood consumption, we calculated the correlation between trimethylamine N-oxide (TMAO) and seafood consumption. TMAO correlated with seafood consumption at rho = 0.07 (N = 726, *P*-value = 0.05) in the younger age group and rho = 0.11(N = 1444, *P*-value = 1.5E-5) in the older group.

### Confectionery consumption is negatively correlated with multiple lipid species

Confectionery consumption positively correlated with three PlsCho species, three Cer species, and SM with hydroxy FA (HFA) C14:1 (hydroxysphingomyelin, SM (OH) C14:1); however, it was negatively correlated with 11 TG and three PC species, as well as PC ae C38:0 and lysoPC a C16:1 (Table 2). Unlike in other food categories, many negative correlations were observed. The negatively correlated lipids either contained omega-3 FA or belonged to the EPA-, DPA-, and DHA-containing cluster, except for lysoPC a C16:1 (Fig. 2). Other food categories were not confounders, as pseudo-correlations among food categories were excluded. Thus, confectionery consumption was independently and negatively correlated with these lipids. Three PlsCho species positively correlated with confectionery consumption had FA with 2–3 double bonds. Given that the human plasma PL content of omega-3 FA with 2–3 double bonds is low (Markey et al., 2017), lipids positively correlated with confectionery consumption were mainly comprised of omega-6 FA.

### Dairy product consumption is positively associated with odd-chain fatty acids and new biomarkers

Dairy product consumption was positively correlated with two SM(OH) species, two PlsCho species, Cer(d16:1/23:0), and lysoPC a C17:0 (Table 2). This study is the first to report the association between dairy product consumption and odd-FA in an Asian population. In addition to odd-FA, SM(OH) C14:1 is known to be correlated with cream consumption, as reported in a previous study (Pallister et al., 2016), while the remaining species are potential new biomarkers of dairy product consumption.

Apart from odd-FA, SM(OH) C14:1, SM(OH) C16:1, PC ae C30:0, and PC ae C36:2 were positively correlated with dairy product consumption. These lipids potentially contain odd-FA in the isobars. In isobars within 0.5 Da, SM(OH) C14:1 contains SM d16:1/17:0 and SM d18:1/15:0, SM(OH) C16:1 contains SM d18:1/17:0, and PlsCho species contain PC that has FA with one less carbon. If the positive correlations between these SM(OH)s and dairy product consumption were due to odd-FA in the isobars, the factor of the correlations of these lipids can be considered to be similar to lysoPC a C17:0 and Cer(d16:1/23:0). Theoretically, a correlation could disappear if controlled by another sharing the same factors. The partial correlation coefficients between dairy product consumption and lipids correlated with dairy product consumption listed in Table 2 were thus recalculated individually with each lipid as an additional covariate. The results showed that the correlation between dairy product consumption and SM(OH) species did not disappear when lysoPC a C17:0 and Cer(d16:1/23:0) were introduced as covariates.

In contrast, the correlation between dairy product consumption and lysoPC a C17:0 was not significant when SM(OH) species were used to control for the correlation (Table 3). Although this was not validated further, the results could suggest that lysoPC a C17:0 correlates with dairy product consumption via SM(OH)s. Furthermore, the correlation between dairy product consumption and SM(OH) C14:1 did not disappear after controlling for other lipids. In contrast, all correlations were suppressed or became negative when SM(OH) C14:1 was added as a covariate. This result suggests that lysoPC a C17:0, along with two PlsCho species, SM(OH) C16:1, and Cer(d16:1/23:0), is correlated with dairy consumption via SM(OH) C14:1.

**Table 3:** Partial Spearman’s rank correlations between dairy products and dairy-product-related lipids

Lipid used as control	Lipid calculated for partial correlation					
	lysoPC a C17:0	PC ae C30:0	PC ae C36:2	SM(OH) C14:1	SM(OH) C16:1	Cer(d16:1/23:0)
lysoPC a C17:0	NA	0.15**/0.13**	0.14**/0.16**	0.26**/0.28**	0.16**/0.19**	0.15**/0.16**
PC ae C30:0	0.12**/0.12**	NA	0.12**/0.16**	0.24**/0.26**	0.13**/0.17**	0.13**/0.15**
PC ae C36:2	0.09*/0.07**	0.12**/0.09**	NA	0.23**/0.23**	0.1**/0.12**	0.13**/0.13**
SM(OH) C14:1	0.06*/0.03	0.05/0	− 0.01/0.01	NA	− 0.13**/ − 0.1**	0.07*/0.08**
SM(OH) C16:1	0.11**/0.08**	0.13**/0.07**	0.1**/0.08**	0.27**/0.24**	NA	0.15**/0.14**
Cer(d16:1/23:0)	0.14**/0.14**	0.15**/0.15**	0.15**/0.19**	0.25**/0.29**	0.17**/0.22**	NA

Partial Spearman’s rank correlation coefficients calculated in the younger and older age group are given separated by a slash (/). Raw *p*-values are shown as: 1–0.05 no symbol, 0.05–0.0016 single asterisk, and < 0.0016 double asterisk. A raw *p*-value of 0.0016 is equal to a Bonferroni adjusted *p*-value of 0.05 in this table

**Fig. 3:**
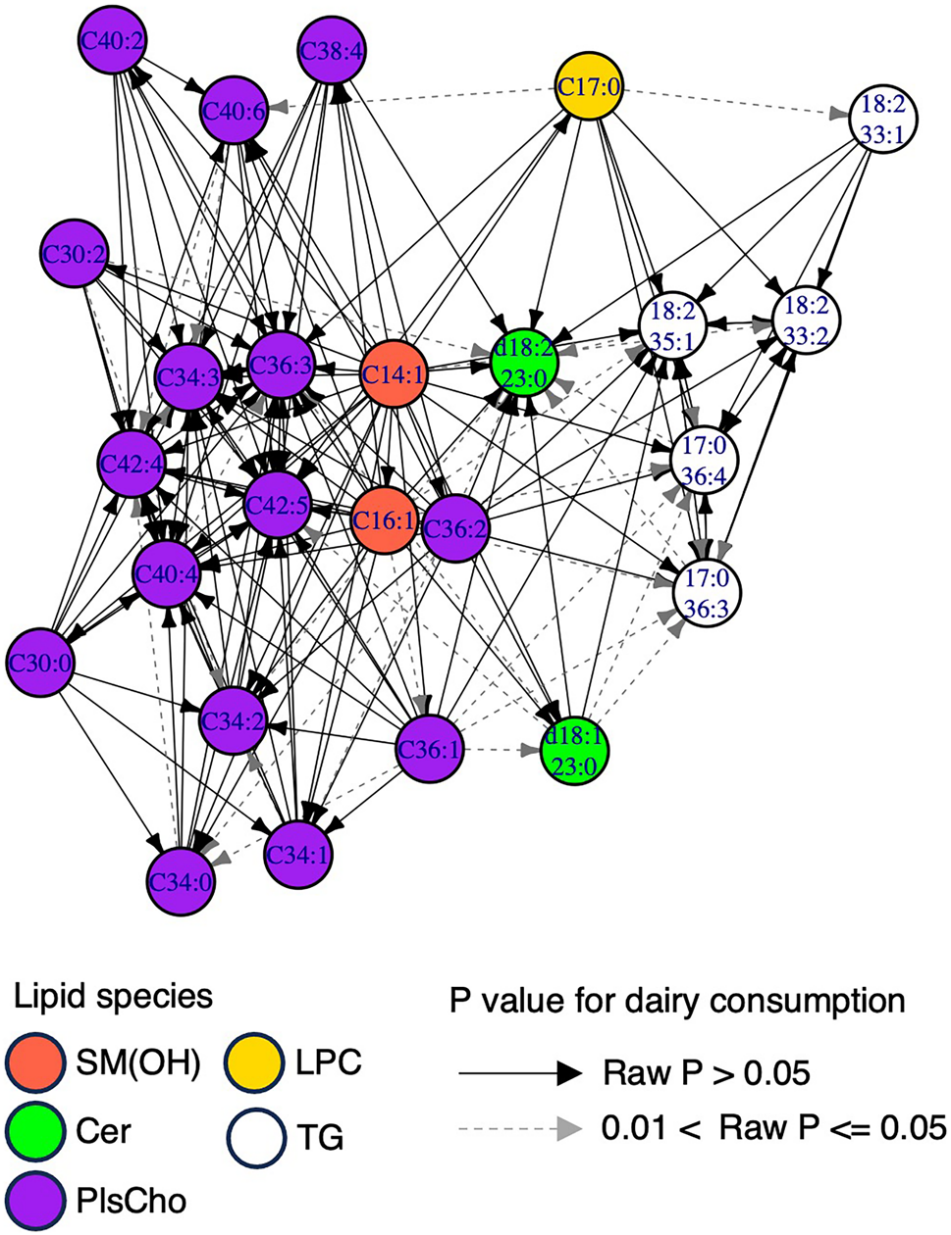
Network showing that the correlations between each odd-FA-containing lipid and dairy product consumption became insignificant when other lipids were added as covariates. The arrows indicate that the correlation between the indicated lipid and dairy consumption became insignificant when the arrow origin lipid was added to the covariates

Because the correlation between dairy product consumption and lysoPC a C17:0 was also suppressed by SM(OH) C14:1, we extended the analysis to the other odd-FA-containing lipid species. SM(OH) and PlsCho species were also included as potential odd-FA-containing lipids based on their isobars. Within the older group, 24 odd-FA-containing lipid species were correlated with dairy consumption at a raw *p*-value of <  = 0.001. Then, we calculated the partial Spearman’s rank sum correlations between these lipids and dairy consumption by introducing one of the other odd-FA-containing lipids as a covariate. We illustrated this result using a network by connecting two lipids by an arrow if the correlation between the arrow end lipid and dairy product consumption became insignificant when the arrow origin lipid was included as a covariate (Fig. 3). Regardless of the inclusion of any odd-FA-containing lipid species in this network, only SM(OH) C14:1 exhibited a significant correlation with dairy consumption. The highest *p*-value was 4.2E-36 by PC ae C36:2. The correlations between dairy consumption and other lipid species, including TG species, were suppressed by adding another odd-FA-containing lipid species. The result suggested that not only PL species but also TG species are correlated with dairy consumption via SM(OH) C14:1.

### Evaluation of the influence of sex, age, and fasting/non-fasting status on the results

Sex differences among lipids and food consumption were evaluated using the Wilcoxon rank-sum test in the younger and older age groups (Online Resources 3 and 4). In both age group, sex differences were observed in various lipids and food consumption (*p* < 0.0001 of the Wilcoxon rank sum test), and the |effect size| was ≥ 0.2. Thus, the effects of sex were assessed via stratified correlation analysis. To assess the consistency of food consumption-lipid correlations identified in this study between males and females, we recalculated all the correlations separately for each sex within the younger and older age groups, and compared the adjusted *p*-values (Online Resources 5). While 17 of the 83 correlations became insignificant in at least one sex at an adjusted *p*-value <  = 0.05 in the older age group (indicated by the superscript letter “a” in Table 2), we verified that the correlations identified in this study exhibited broad consistency, except in the younger male group. This consistency could not be assessed in the younger age male group due to the small number of samples. Similarly, the consistency of the correlations by age group was evaluated in males and females (Online Resources 5). Eleven correlations became insignificant in at least one age group at an adjusted *p*-value <  = 0.05 in females (indicated by the superscript letter “b” in Table 2). We also confirmed the consistency of fasting/non-fasting status. Seventeen correlations, which were not significant in at least one of the fasting/non-fasting groups, are shown with the superscript letter “c” in Table 2.

## Discussion

We conducted correlation analyses between food consumption habits and plasma lipid concentrations in 4,032 Japanese individuals. Although the correlation between TMAO and seafood consumption was uncertain in the younger age group in this study, the results confirmed that our omics panel and food category consumption panel were suitable for use in identifying dietary lipid biomarkers, as seafood consumption and omega-3 FA were validated within the results. Apart from omega-3 FA, SL containing C26:1 was also validated as a seafood consumption marker. Because SM and Cer are lipid subclasses that are poorly absorbed from dietary SL (Nilsson, 1968), these correlations may be due to C26:1. Approximately 0.1% of the total FA of herring is C26:1 (Linko & Karinkanta, 1970; Nilsson, 1968); however, this component has not been isolated from non-seafood sources. According to the Standard Tables of Food Composition in Japan (Resource Council et al., 2002), monounsaturated very-long-chain FA are uniquely present in fish; a 100-g edible portion of fish contains dozens of milligrams of C22:1 and C24:1, while beef, pork, and chicken do not contain these lipids. If this trend extends to C26:1, it may be concluded that SM C26:1 and Cer(d18:1/26:1) are positively correlated with seafood consumption, as C26:1 is an FA marker of seafood.

To the best of our knowledge, this is the first study to validate dairy product consumption and odd-FA levels in an Asian population. Although an association between dairy product consumption and odd-FA has been observed in European and Australasian populations, this association has not been previously reported in any Japanese population. The low consumption of dairy products by Japanese individuals could be one reason; males in European and Australasian countries consume 300–500 g/day of dairy products, while participants in this study consumed less than 200 g/day (Albani et al., 2017; Golley & Hendrie, 2014; Warensjö et al., 2010). Given this context, this study used large-scale data to confirm that dairy product consumption is associated with odd-FA in the Japanese population. Furthermore, the results of this study suggest a correlation between odd-FA-containing lipid species and dairy product consumption via SM. Partial correlation analysis may be useful in determining the specific transport pathway for a given FA, but this has not been validated. However, we speculate that SM could be a carrier of odd-FA to LPC and PC.

Although all correlations between dairy product consumption and lipids were possibly due to odd-FA based on the potential isobars, gut lactic bacteria are worthy of mention in this context, because SM(OH) species possibly contain HFA as an intermediate product of the hydrogenation of polyunsaturated FA by gut lactic acid bacteria (Morito et al., 2019), the population of which is increased by dairy product consumption (Aslam et al., 2020). HFA generated by gut microbes are absorbed by the host and introduced into GPL (Morito et al., 2019); however, whether HFA are introduced into SL remains unknown.

The study provides several beneficial insights into daily habits. Seafood consumption and confectionery consumption have opposite effects on omega-3 FA, the beneficial effects of which have been reported in many studies and include reduction in mortality (Harris et al., 2021; Zhuang et al., 2019), lower cardiovascular risk (Bernasconi et al., 2021a, 2021b; Bernasconi et al., 2021a, 2021b), a lower risk of preterm birth (Middleton et al., 2018), regulation and resolution of inflammation (Calder, 2017), and regulation of the normal immune response (Gutiérrez et al., 2019). Although the mechanism of the negative association between confectionery consumption and omega-3 FA is unclear, if confectionery consumption provides omega-6 FA, it could negatively associate with omega-3 FA because a negative correlation between omega-3 FA and omega-6 FA as biomarkers of dietary FA intake has been previously reported (Astorg et al., 2008; Friesen & Innis, 2010; Ma et al., 1995). Our results in terms of omega-3 FA suggested that seafood consumption continues to be a beneficial dietary habit in the Japanese population and that caution should be exercised in the confectionery consumption. The beneficial effects of odd-FA have been suggested for Japanese populations (Kurotani et al., 2017; Maruyama et al., 2008). Moreover, a recent study proposed C15:0 as an essential FA because C15:0 is not readily made by the body, and lower body levels of C15:0 have been associated with poorer cardiometabolic function. C15:0 has been demonstrated to have beneficial and pleiotropic activities directly related to cardiometabolic, immune, and liver health (Dornan et al., 2021; Venn-Watson et al., 2020). A recent study highlighted that the relevant activities of C15:0 are comparable to or even surpasses that of EPA, an approved therapeutic agent (Venn-Watson et al., 2022). Understanding the influence of dietary habits on odd-FA represents a direct line of evidence linking diet to disease. The results of our study suggest that dairy product consumption is effective for the consumption of odd-FA in the Japanese population. As a part of the significant goal of the TMM CommCohort Study to provide knowledge about the associations between dietary habits and health to the public, we believe that the present study provides fundamental knowledge. Individual food consumption and metabolite data used in this study are available upon request after approval of the Ethical Committee and the Materials and Information Distribution Review Committee of Tohoku Medical Megabank Organization.

Additionally, this study demonstrates the potential use of the MxP Quant 500 Kit for evaluating lipid concentrations, specifically omega-3 and odd-FA, which are implicated in various diseases. This suggests that the MxP Quant 500 Kit may serve as a tool for assessing lipid concentrations as an intermediary indicator of diet-disease relationships. The research also suggests the prospect of identifying crucial FA biomarkers within sphingolipids. Notably, SM(OH) C14:1 emerged as the most effective biomarker for odd-FA, and dietary traces of very long-chain FA, such as C26:1, were detected in SL. However, the inclusion of isobars often complicated the interpretation of these results.γ

Possible future work may reveal the health effects of the associations observed in this study. The TMM CommCohort Study collects information on dietary habits and other data, including medical history. Using these data, we expect to learn how metabolites behave as an intermediate phenotype between diet and health.

One limitation of this study is that our metabolomics platform did not identify exact lipid structures but included isobars and isomers. We identified lipid species in a preliminary manner based on acyl residues, sums of double bonds, and known FA compositions in human plasma. In the dietary data, FFQ is a questionnaire applied across a year, which is less accurate than more precise measurement approaches such as weighed food records. Another limitation of this study is that the various covariates were adjusted in the model. It should be noted that our stratified analyses for sex, age group, and fasting/non-fasting status may have increased the risk for potential false-positive correlations.

In summary, we identified 84 significant correlations between 6 of 15 food categories and plasma lipid concentrations in nine subclasses. For the first time, we confirmed a correction between dairy product consumption and odd-FA in an Asian population. We identified correlations in lipid subclasses that have not often been targeted in previous studies, including PlsCho, Cer, and SM. Our results make a significant contribution to understanding the effect of dietary habits on plasma lipid concentrations.

### Supplementary Information

Below is the link to the electronic supplementary material.

Supplementary file1 (DOCX 458 kb)
