# Supplementary material for: Dietary habits and plasma lipid concentrations in a general Japanese population
Source: Metabolomics. 2024 Mar 5;20(2):34. doi: 10.1007/s11306-024-02087-1 (PMC10914877; doi:10.1007/s11306-024-02087-1)
Supplement: Supplementary file 1 — Supplementary file1 (DOCX 458 kb) [file 11306_2024_2087_MOESM1_ESM.docx]

Supplementary Information

**Dietary habits and plasma lipid concentrations in a general Japanese population**

*Metabolomics*

Mitsuharu Sato^1,7^, Eiji Hishinuma^1,2,7^, Naomi Matsukawa^1^, Yoshiko Shima^1^, Daisuke Saigusa^1,3^, Ikuko N. Motoike^1,6^, Mana Kogure^1^, Naoki Nakaya^1^, Atsushi Hozawa^1,4^, Shinichi Kuriyama^1,4,5^, Masayuki Yamamoto^1^, Seizo Koshiba^1,2^, and Kengo Kinoshita^1,2,6,8,9,*^

^1^ Tohoku Medical Megabank Organization, Tohoku University, 2-1, Seiryo-machi, Aoba-ku, Sendai, Miyagi, 980-8573, Japan

^2^ Advanced Research Center for Innovations in Next-Generation Medicine, Tohoku University, 2-1, Seiryo-machi, Aoba-ku, Sendai, Miyagi, 980-8573, Japan

^3^ Laboratory of Biomedical and Analytical Sciences, Faculty of Pharma-Science, Teikyo University, 2-11-1, Kaga, Itabashi-ku, Tokyo, 173-8605, Japan

^4^ Graduate School of Medicine, Tohoku University, 2-1 Seiryo-machi, Aoba-ku, Sendai, Miyagi, 980-8575, Japan

^5^ International Research Institute of Disaster Science, Tohoku University, 2-1 Seiryo-machi, Aoba-ku, Sendai, Miyagi, 980-8573, Japan

^6^ Graduate School of Information Sciences, Tohoku University, 6-3-09 Aramaki Aza-Aoba, Aoba-ku, Sendai, Miyagi, 980-8579, Japan

^7^ These authors contributed equally

^8^ Senior author

^9^ Lead contact

^*^ Correspondence: [kengo@tohoku.ac.jp](mailto:kengo@tohoku.ac.jp)

**
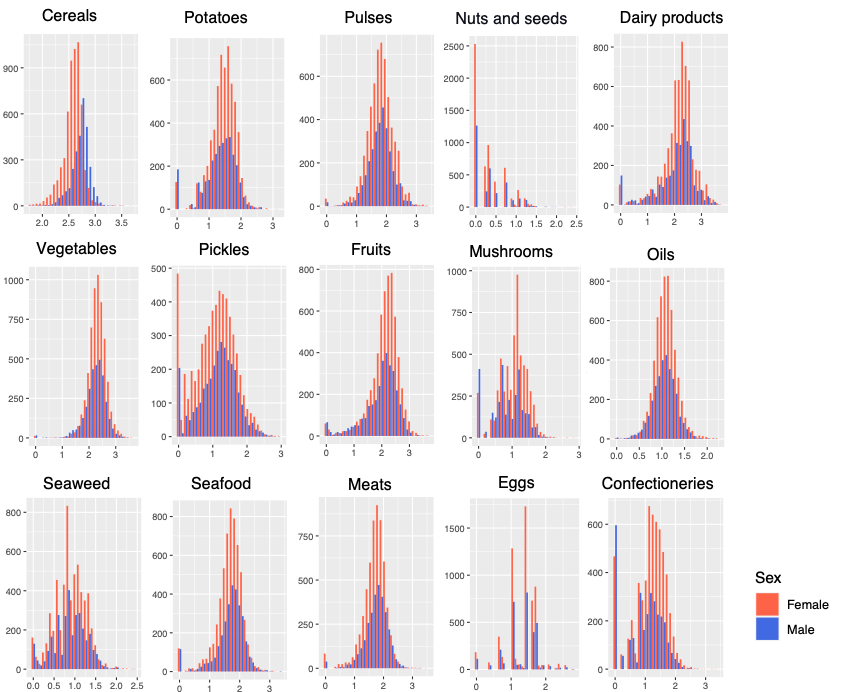
**

**Online Resource 1 Distribution of daily consumption volumes for each food category** Consumption volumes were log transformed. The histograms include both age groups in the discovery dataset shown separately by sex. Males and females correspond to blue and red, respectively.

**Online Resource 2 Partial Spearman’s correlation coefficients among food groups**

|  | Cereals | Potatoes | Pulses | Nuts and seeds | Vegetables | Pickles | Fruits | Mushrooms | Seaweed | Seafood | Meat | Eggs | Dairy |  |
| --- | --- | --- | --- | --- | --- | --- | --- | --- | --- | --- | --- | --- | --- | --- |
|  |  |  |  |  |  |  |  |  |  |  |  |  |  |  |
| Potatoes | -0.01 |  |  |  |  |  |  |  |  |  |  |  |  |  |
| Pulses | -0.11 | 0.23 |  |  |  |  |  |  |  |  |  |  |  |  |
| Nuts and seeds | -0.04 | 0.12 | 0.11 |  |  |  |  |  |  |  |  |  |  |  |
| Vegetables | -0.09 | 0.38 | 0.21 | 0.07 |  |  |  |  |  |  |  |  |  |  |
| Pickles | 0.05 | 0.18 | 0.07 | 0.07 | 0.41 |  |  |  |  |  |  |  |  |  |
| Fruits | -0.06 | 0.25 | 0.12 | 0.12 | 0.35 | 0.24 |  |  |  |  |  |  |  |  |
| Mushrooms | -0.07 | 0.40 | 0.22 | 0.11 | 0.32 | 0.06 | 0.21 |  |  |  |  |  |  |  |
| Seaweed | -0.04 | 0.37 | 0.25 | 0.12 | 0.30 | 0.14 | 0.19 | 0.41 |  |  |  |  |  |  |
| Seafood | -0.07 | 0.19 | 0.12 | 0.09 | 0.20 | 0.15 | 0.11 | 0.23 | 0.25 |  |  |  |  |  |
| Meat | -0.11 | 0.04 | -0.06 | 0.04 | -0.03 | -0.03 | -0.05 | 0.06 | 0.04 | 0.20 |  |  |  |  |
| Eggs | -0.04 | 0.08 | 0.04 | 0.00 | 0.08 | 0.03 | 0.00 | 0.07 | 0.07 | 0.08 | 0.11 |  |  |  |
| Dairy | -0.21 | 0.00 | 0.02 | -0.01 | 0.03 | -0.05 | 0.09 | 0.01 | 0.04 | -0.03 | -0.11 | 0.08 |  |  |
| Confectionery | -0.04 | 0.14 | 0.02 | 0.28 | 0.03 | 0.04 | 0.15 | 0.13 | 0.07 | 0.07 | 0.05 | 0.02 | 0.01 |  |

Stronger positive correlations correspond to a more intense red color, whereas stronger negative correlations correspond to a more intense blue color. Data for all participants in both age groups in the discovery dataset are included.

**Online Resource 3 Sex differences among the lipids that significantly correlated with food consumption in this study**

| Lipid Name | Young | | | | | | | Old | | | | | | |
| --- | --- | --- | --- | --- | --- | --- | --- | --- | --- | --- | --- | --- | --- | --- |
|  | Female | | Male | | Sex differences | Effect size |  | Female | | Male | | Sex differences | Effect size |  |
|  | N | Shapiro-wilk test | N | Shapiro-wilk test |  |  |  | N | Shapiro-wilk test | N | Shapiro-wilk test |  |  |  |
| lysoPC a C16:1 | 951 | 0.000 | 285 | 0.000 | 0.000 | -0.471 | * | 1567 | 9.81E-08 | 1075 | 0.003 | 0.000 | -0.301 | * |
| lysoPC a C17:0 | 951 | 0.004 | 285 | 0.636 | 0.006 | -0.193 |  | 1567 | 0.000 | 1075 | 0.009 | 0.000 | 0.338 | * |
| PC aa C28:1 | 1010 | 0.661 | 287 | 0.533 | 0.000 | 0.331 | * | 1670 | 0.090 | 1080 | 0.912 | 0.000 | 0.762 | * |
| PC aa C32:1 | 1010 | 0.000 | 287 | 0.001 | 0.356 | -0.099 |  | 1670 | 1.66E-06 | 1080 | 0.001 | 0.265 | -0.054 |  |
| PC aa C36:0 | 1003 | 0.000 | 282 | 0.000 | 0.905 | 0.053 |  | 1665 | 0.000 | 1076 | 0.000 | 0.715 | -0.018 |  |
| PC aa C36:3 | 1010 | 0.271 | 287 | 0.088 | 0.705 | -0.021 |  | 1670 | 0.000 | 1080 | 0.000 | 0.000 | 0.479 | * |
| PC aa C36:5 | 1010 | 0.000 | 287 | 0.524 | 0.000 | -0.241 | * | 1670 | 0.227 | 1080 | 0.146 | 0.000 | -0.323 | * |
| PC aa C36:6 | 1010 | 0.471 | 287 | 0.195 | 0.308 | -0.063 |  | 1670 | 0.003 | 1080 | 0.100 | 0.460 | -0.029 |  |
| PC aa C38:0 | 1010 | 0.925 | 287 | 0.068 | 0.000 | 0.341 | * | 1670 | 0.017 | 1080 | 0.027 | 0.000 | 0.366 | * |
| PC aa C38:5 | 1010 | 0.000 | 287 | 0.033 | 0.014 | -0.171 |  | 1670 | 0.060 | 1080 | 0.460 | 0.698 | 0.016 |  |
| PC aa C38:6 | 1010 | 0.008 | 287 | 0.001 | 0.287 | 0.125 |  | 1670 | 0.000 | 1080 | 0.026 | 0.000 | 0.168 |  |
| PC aa C40:3 | 1010 | 0.000 | 287 | 0.001 | 0.457 | -0.055 |  | 1669 | 0.000 | 1080 | 0.000 | 0.043 | 0.078 |  |
| PC aa C40:6 | 1010 | 0.016 | 287 | 0.011 | 0.024 | -0.111 |  | 1670 | 0.000 | 1080 | 0.008 | 0.000 | 0.190 |  |
| PC aa C42:0 | 1010 | 0.325 | 287 | 0.663 | 0.000 | 0.577 | * | 1670 | 0.251 | 1080 | 0.140 | 0.000 | 0.233 | * |
| PC aa C42:1 | 1010 | 0.643 | 287 | 0.304 | 0.000 | 0.438 | * | 1670 | 0.121 | 1080 | 0.194 | 0.000 | 0.323 | * |
| PC aa C42:2 | 1010 | 0.020 | 287 | 0.019 | 0.866 | -0.014 |  | 1670 | 0.001 | 1080 | 0.956 | 0.232 | -0.042 |  |
| PC aa C42:5 | 1010 | 0.000 | 287 | 0.000 | 0.450 | 0.031 |  | 1670 | 0.000 | 1080 | 0.000 | 0.792 | 0.020 |  |
| PC aa C42:6 | 1004 | 0.000 | 287 | 0.000 | 0.314 | 0.055 |  | 1656 | 0.000 | 1074 | 0.000 | 0.001 | 0.120 |  |
| PC ae C30:0 | 1006 | 0.026 | 286 | 0.047 | 0.000 | 0.652 | * | 1639 | 0.724 | 1076 | 0.677 | 0.000 | 0.296 | * |
| PC ae C30:1 | 887 | 0.000 | 284 | 0.000 | 0.000 | 0.369 | * | 1592 | 0.000 | 1075 | 0.005 | 0.000 | 0.134 |  |
| PC ae C32:2 | 1010 | 0.007 | 287 | 0.173 | 0.000 | 0.607 | * | 1670 | 0.005 | 1080 | 0.127 | 0.000 | 0.415 | * |
| PC ae C34:0 | 1010 | 0.654 | 287 | 0.098 | 0.000 | 0.336 | * | 1670 | 0.373 | 1080 | 0.759 | 0.000 | 0.292 | * |
| PC ae C34:2 | 1010 | 0.071 | 287 | 0.379 | 0.000 | 0.729 | * | 1670 | 0.053 | 1080 | 0.982 | 0.000 | 0.655 | * |
| PC ae C36:0 | 1010 | 0.000 | 287 | 0.116 | 0.786 | -0.045 |  | 1670 | 0.000 | 1080 | 0.464 | 0.000 | -0.199 |  |
| PC ae C36:2 | 1010 | 0.000 | 287 | 0.919 | 0.000 | 0.609 | * | 1670 | 0.560 | 1080 | 0.000 | 0.000 | 0.828 | * |
| PC ae C36:3 | 1010 | 0.004 | 287 | 0.995 | 0.000 | 0.644 | * | 1670 | 0.087 | 1080 | 0.067 | 0.000 | 0.672 | * |
| PC ae C36:4 | 1010 | 0.203 | 287 | 0.006 | 0.127 | 0.076 |  | 1670 | 0.448 | 1080 | 0.511 | 0.000 | 0.356 | * |
| PC ae C38:0 | 1010 | 0.707 | 287 | 0.735 | 0.766 | -0.011 |  | 1670 | 0.035 | 1080 | 0.175 | 0.776 | 0.019 |  |
| PC ae C38:4 | 1010 | 0.274 | 287 | 0.759 | 0.000 | 0.311 | * | 1670 | 0.713 | 1080 | 0.256 | 0.000 | 0.646 | * |
| PC ae C38:5 | 1010 | 0.446 | 287 | 0.064 | 0.079 | 0.104 |  | 1670 | 0.524 | 1080 | 0.265 | 0.000 | 0.299 | * |
| PC ae C38:6 | 1010 | 0.280 | 287 | 0.834 | 0.096 | 0.112 |  | 1670 | 0.984 | 1080 | 0.817 | 0.002 | 0.128 |  |
| PC ae C40:2 | 1010 | 0.102 | 287 | 0.440 | 0.000 | 0.307 | * | 1670 | 0.977 | 1080 | 0.322 | 0.000 | 0.565 | * |
| PC ae C40:5 | 1010 | 0.022 | 287 | 0.138 | 0.000 | 0.262 | * | 1670 | 0.020 | 1080 | 0.412 | 0.000 | 0.300 | * |
| PC ae C40:6 | 1010 | 0.898 | 287 | 0.211 | 0.000 | 0.43 | * | 1670 | 0.106 | 1080 | 0.043 | 0.000 | 0.550 | * |
| SM (OH) C14:1 | 1010 | 0.063 | 287 | 0.857 | 0.000 | 0.626 | * | 1670 | 0.065 | 1080 | 0.203 | 0.000 | 0.927 | * |
| SM (OH) C16:1 | 1010 | 0.014 | 287 | 0.670 | 0.000 | 0.518 | * | 1670 | 0.237 | 1080 | 0.001 | 0.000 | 0.866 | * |
| SM C26:1 | 497 | 0.118 | 145 | 0.594 | 0.035 | -0.215 |  | 438 | 0.129 | 488 | 0.138 | 0.006 | -0.154 |  |
| DHA | 829 | 0.000 | 268 | 0.367 | 0.039 | 0.056 |  | 1474 | 0.000 | 1050 | 0.000 | 0.659 | -0.050 |  |
| EPA | 735 | 0.002 | 258 | 0.018 | 0.583 | 0.039 |  | 1338 | 0.000 | 1009 | 0.000 | 0.844 | -0.021 |  |
| Cer(d16:1/23:0) | 849 | 0.003 | 262 | 0.690 | 0.307 | 0.051 |  | 1378 | 0.792 | 913 | 0.104 | 0.000 | 0.411 | * |
| Cer(d18:1/22:0) | 1007 | 0.620 | 287 | 0.667 | 0.052 | -0.127 |  | 1619 | 0.150 | 1078 | 0.009 | 0.000 | 0.452 | * |
| Cer(d18:1/26:1) | 690 | 0.000 | 222 | 0.000 | 0.009 | -0.098 |  | 1010 | 0.000 | 744 | 0.000 | 0.000 | -0.189 |  |
| Cer(d18:2/22:0) | 1007 | 0.019 | 287 | 0.207 | 0.110 | 0.115 |  | 1657 | 0.000 | 1078 | 0.000 | 0.000 | 0.601 | * |
| Cer(d18:2/24:0) | 1008 | 0.121 | 287 | 0.249 | 0.000 | -0.25 | * | 1661 | 0.006 | 1080 | 0.101 | 0.000 | 0.489 | * |
| CE(20:5) | 880 | 0.607 | 243 | 0.990 | 0.464 | -0.042 |  | 1521 | 0.000 | 999 | 0.000 | 0.072 | -0.055 |  |
| CE(22:5) | 890 | 0.000 | 279 | 0.000 | 0.237 | 0.127 |  | 1536 | 0.000 | 1050 | 0.000 | 0.000 | 0.286 | * |
| CE(22:6) | 970 | 0.000 | 269 | 0.009 | 0.029 | 0.167 |  | 1653 | 0.000 | 1070 | 0.000 | 0.000 | 0.295 | * |
| Hex3Cer(d18:1_22:0) | 691 | 0.027 | 240 | 0.501 | 0.000 | 0.578 | * | 1082 | 0.373 | 800 | 0.400 | 0.000 | 0.561 | * |
| TG(16:0_38:6) | 679 | 0.000 | 221 | 0.043 | 0.000 | -0.782 | * | 1031 | 0.006 | 810 | 0.327 | 0.000 | -0.555 | * |
| TG(16:0_38:7) | 527 | 0.001 | 144 | 0.898 | 0.000 | -0.633 | * | 595 | 0.415 | 578 | 0.024 | 0.000 | -0.395 | * |
| TG(16:0_40:7) | 685 | 0.656 | 206 | 0.383 | 0.000 | -0.388 | * | 824 | 0.006 | 643 | 0.188 | 0.000 | -0.416 | * |
| TG(16:0_40:8) | 836 | 0.003 | 250 | 0.195 | 0.000 | -0.469 | * | 1237 | 0.000 | 904 | 0.000 | 0.000 | -0.330 | * |
| TG(16:1_34:3) | 849 | 0.000 | 264 | 0.895 | 0.000 | -0.47 | * | 1530 | 0.154 | 1027 | 0.958 | 0.000 | -0.212 | * |
| TG(16:1_38:5) | 871 | 0.205 | 262 | 0.813 | 0.000 | -0.726 | * | 1506 | 0.887 | 994 | 0.027 | 0.000 | -0.416 | * |
| TG(18:0_38:6) | 975 | 0.025 | 286 | 0.005 | 0.000 | -0.577 | * | 1623 | 0.009 | 1068 | 0.004 | 0.000 | -0.160 |  |
| TG(18:1_38:6) | 1008 | 0.000 | 286 | 0.384 | 0.000 | -0.661 | * | 1645 | 0.303 | 1064 | 0.571 | 0.000 | -0.386 | * |
| TG(18:1_38:7) | 862 | 0.002 | 262 | 0.015 | 0.000 | -0.449 | * | 1600 | 0.000 | 1044 | 0.000 | 0.000 | -0.191 |  |
| TG(18:2_38:6) | 992 | 0.019 | 283 | 0.052 | 0.000 | -0.478 | * | 1634 | 0.188 | 1062 | 0.236 | 0.000 | -0.346 | * |
| TG(20:5_34:1) | 995 | 0.016 | 285 | 0.744 | 0.000 | -0.732 | * | 1663 | 0.015 | 1074 | 0.004 | 0.000 | -0.501 | * |
| TG(20:5_34:2) | 979 | 0.230 | 287 | 0.672 | 0.000 | -0.592 | * | 1545 | 0.000 | 1074 | 0.000 | 0.000 | -0.379 | * |
| TG(20:5_36:2) | 924 | 0.262 | 263 | 0.217 | 0.000 | -0.553 | * | 1512 | 0.000 | 1051 | 0.000 | 0.000 | -0.289 | * |
| TG(20:5_36:3) | 943 | 0.128 | 281 | 0.002 | 0.000 | -0.39 | * | 1600 | 0.000 | 1056 | 0.000 | 0.000 | -0.157 |  |
| TG(22:5_34:1) | 952 | 0.001 | 274 | 0.354 | 0.000 | -0.669 | * | 1314 | 0.022 | 899 | 0.452 | 0.000 | -0.411 | * |
| TG(22:5_34:2) | 991 | 0.245 | 286 | 0.279 | 0.000 | -0.575 | * | 1568 | 0.080 | 1071 | 0.401 | 0.000 | -0.328 | * |
| TG(22:6_32:0) | 929 | 0.008 | 269 | 0.145 | 0.000 | -0.658 | * | 1642 | 0.127 | 1068 | 0.000 | 0.000 | -0.527 | * |
| TG(22:6_32:1) | 720 | 0.613 | 228 | 0.005 | 0.000 | -0.345 | * | 1069 | 0.000 | 840 | 0.000 | 0.000 | -0.436 | * |
| TG(22:6_34:1) | 1009 | 0.001 | 285 | 0.000 | 0.000 | -0.47 | * | 1668 | 0.000 | 1079 | 0.000 | 0.000 | -0.475 | * |
| TG(22:6_34:2) | 1004 | 0.448 | 285 | 0.061 | 0.000 | -0.474 | * | 1670 | 0.000 | 1078 | 0.000 | 0.000 | -0.425 | * |
| TG(22:6_34:3) | 665 | 0.001 | 198 | 0.001 | 0.000 | -0.316 | * | 1303 | 0.000 | 885 | 0.000 | 0.000 | -0.337 | * |

Sex difference was estimated by using Wilcoxon rank-sum test. The values for the test were the log-transformed lipid concentration. Effect size which was subtract male from female was calculated by using Cohen’s d (Cohen 2013) .

Sex differences Wilcoxon rank-sum test p-value <= 0.001 and | Cohen’s d| >= 0.2 were shown by *.

**Online Resource 4 Sex differences among food consumption**

| Food Name | Young | | | | | | Old | | | | | |
| --- | --- | --- | --- | --- | --- | --- | --- | --- | --- | --- | --- | --- |
|  | Female | | Male | | Sex differences | Effect size | Female | | Male | | Sex differences | Effect size |
|  | N | Shapiro-wilk test | N | Shapiro-wilk test |  |  | N | Shapiro-wilk test | N | Shapiro-wilk test |  |  |
| Cereals | 1010 | 0 | 287 | 0 | **0** | **-0.70** | 1670 | 0 | 1080 | 0 | **0** | **-0.72** |
| Potatoes | 1010 | 0 | 287 | 0 | **0** | **0.42** | 1670 | 0 | 1080 | 0 | 0 | 0.41 |
| Pulses | 1010 | 0 | 287 | 0.006 | **0** | **0.26** | 1670 | 0 | 1080 | 0 | 0 | 0.17 |
| Nuts and seeds | 1010 | 0 | 287 | 0 | 0.278 | -0.11 | 1670 | 0 | 1080 | 0 | 0.94 | 0.01 |
| Vegetables | 1010 | 0 | 287 | 0 | **0** | **0.38** | 1670 | 0 | 1080 | 0 | **0** | **0.46** |
| Pickles | 1010 | 0 | 287 | 0 | 0.169 | -0.07 | 1670 | 0 | 1080 | 0 | 0.42 | 0.03 |
| Fruits | 1010 | 0 | 287 | 0 | **0** | **0.35** | 1670 | 0 | 1080 | 0 | **0** | **0.47** |
| Mushrooms | 1010 | 0 | 287 | 0 | **0** | **0.61** | 1670 | 0 | 1080 | 0 | **0** | **0.58** |
| Seaweed | 1010 | 0 | 287 | 0 | 0.038 | 0.16 | 1670 | 0 | 1080 | 0 | 0.001 | 0.11 |
| Seafood | 1010 | 0 | 287 | 0 | 0.968 | 0.08 | 1670 | 0 | 1080 | 0 | 0.09 | 0.13 |
| Meat | 1010 | 0 | 287 | 0.033 | 0.001 | -0.22 | 1670 | 0 | 1080 | 0 | 0.98 | -0.01 |
| Eggs | 1010 | 0 | 287 | 0 | 0.492 | 0.10 | 1670 | 0 | 1080 | 0 | 0.76 | 0.02 |
| Dairy | 1010 | 0 | 287 | 0 | **0** | **0.34** | 1670 | 0 | 1080 | 0 | **0** | **0.36** |
| Fats and oils | 1010 | 0 | 287 | 0.034 | 0.044 | 0.15 | 1670 | 0 | 1080 | 0 | **0** | **0.33** |
| Confectionery | 1010 | 0 | 287 | 0 | **0** | **0.46** | 1670 | 0 | 1080 | 0 | **0** | **0.47** |

Sex difference was estimated by using Wilcoxon rank-sum test. The values for the test were the log-transformed food consumption. Effect size which was subtract male from female was calculated by using Cohen’s d (Cohen 2013).

**Online Resource 5. Assessing the consistency of food-lipid correlations in male and female as well as younger and older age group.**

**Online Resource 6. Assessing the consistency of food-lipid correlations in fasting and non-fasting. The correlations were calculated by using only older age group.**
